# Growth Performance and Fitness Analysis of Backcross Progenies of Herbicide-Tolerant Transgenic Soybean and Wild Soybean

**DOI:** 10.3390/plants15172730

**Published:** 2026-09-07

**Authors:** Li Zhang, Xiaojing Wang, Laipan Liu, Qi Yu, Wenjing Shen, Zhixiang Fang, Yuliang Zhang, Biao Liu

**Affiliations:** 1 Key Laboratory on Biosafety of Nanjing Institute of Environmental Sciences, Ministry of Ecology and Environment, Nanjing 210042, China; feiniao8897@126.com (L.Z.); liulaipan@163.com (L.L.); 15968821722@163.com (Q.Y.); swj@nies.org (W.S.); zxfang23@126.com (Z.F.); 2 Inner Mongolia Innovation Center of Biological Breeding Technology, Hinngan League Academy of Agricultural and Animal Husbandry Sciences, Ulanhot 137400, China; wxiaojing0216@163.com; 3 Key Laboratory of Nanfan Biotech Breeding, Ministry of Agriculture and Rural Affairs, Sanya Research Institute, Chinese Academy of Tropical Agricultural Sciences, Sanya 572525, China; zhangyuliang@catasitbb.cn

**Keywords:** GM4032, wild soybean, backcross progeny, gene flow, fitness, ecological risk

## Abstract

Gene escape from genetically modified (GM) soybean may have ecological and genetic effects on wild populations. To assess the potential risks associated with backcrossing GM soybean with wild soybean, seed dormancy, inheritance of herbicide resistance, and plant growth in F_1_, BC_1_F_1_, and BC_2_F_1_ lines were comprehensively analyzed. The results showed that, compared with those of wild soybean, the seeds of BC_1_F_1_ and BC_2_F_1_ also exhibited dormancy, albeit at significantly lower levels. BC_1_F_1_ and BC_2_F_1_ showed reduced biomass (by 6–7%; *p* > 0.05) and inferior reproductive performance, with the numbers of pods, seeds, and filled seeds per plant decreasing by 27.02–29.57% (*p* < 0.05). However, glyphosate spraying tests demonstrated that 46.3% of the BC_1_F_1_ plants and 30.6% of the BC_2_F_1_ plants were resistant to glyphosate. Moreover, exogenous protein assay results confirmed that the exogenous gene was stably inherited in these plants, which confers a significant growth advantage under selection pressure. These findings provide new scientific evidence for assessing the ecological risks associated with the environmental release of GM soybean.

## 1. Introduction

Soybean (*Glycine max* (L.) Merrill) is one of the most economically and socially important crops in China [1,2,3]. Given the limited arable land in the country, green scientific and technological innovations are needed to increase soybean production [4]. The introduction of exogenous genes can confer traits such as pest resistance, herbicide tolerance, and high yield in recipient soybean plants [5], thereby simplifying cultivation management and improving production efficiency; this has played a significant role in driving the global soybean industry [6]. By the end of 2021, 40 genetically modified (GM) soybean transformation events had received commercial cultivation approval worldwide [7].

Although GM soybeans offer considerable agronomic benefits, they inevitably interact with the surrounding environment during their growth, posing potential risks to the ecological environment. These risks include effects on target and non-target organisms [8,9], the potential for GM soybeans to become weedy either directly or through gene flow with wild relatives, and impacts on soil ecosystems and biogeochemical cycles [10,11,12,13]. Therefore, before commercial cultivation, GM soybeans must be subjected to a systematic and in-depth environmental safety assessment.

Gene flow from GM soybeans to their wild relatives has remained among the main ecological concerns that must be addressed in the event of their commercial release [14,15,16]. Wild soybean (*Glycine soja*), the ancestor of cultivated soybean, is a unique species endemic to East Asia [17,18,19]. As the center of the origin and diversification of wild soybean, China harbors abundant and widely distributed wild soybean germplasm resources [20,21]. Wild soybeans are predominantly found in low-humidity environments, such as field edges, roadsides, banks and ditches, and frequently grow in close association with cultivated soybean and corn fields [22]. The high spatial distribution overlap between wild and cultivated soybeans provides favorable conditions for gene flow. Consequently, once GM soybeans are released into the environment, gene flow between them and wild soybean populations would be inevitable.

At present, research on gene flow from GM soybeans to wild soybeans has mainly analyzed ecological risks associated with hybrid offspring of GM and wild soybeans [15,23,24]. Similarly to hybridization, hybrid F_1_ generations carrying resistance genes can also be backcrossed with the maternal parent. Moreover, because backcross offspring retain a higher proportion of wild soybean genetic information than hybrids, they may pose even greater ecological risks than hybrid offspring. However, relatively few studies have investigated the ecological and environmental risks of backcross offspring derived from GM and wild soybeans. Only Liu et al. [16] evaluated the environmental risks of the BC_1_F_1_ backcross generation under potted conditions; nevertheless, their study focused primarily on the growth and yield of backcross offspring, with limited attention paid to the seeds.

Seeds serve as important vehicles for the dissemination of exogenous genes [12,25,26], and the seeds’ size, quality, and dormancy can affect seed dispersal [27,28]. During the long process of domestication, the seed dormancy and hardness of soybean gradually decreased, whereas wild soybean maintained a high level of seed dormancy owing to the mechanical constraints imposed by its seed coat. After backcrossing between GM and wild soybeans, the backcross offspring may inherit a combination of traits from both parental lines. If the seeds of backcross offspring exhibit no or low dormancy, they will germinate immediately upon reaching the soil, and the resulting seedlings will die during the cold winter season; in this case, the probability of exogenous gene spread via seeds is relatively low. Conversely, if the seeds of backcross offspring exhibit a level of dormancy comparable with that of wild soybean, they can persist in the soil seed bank and germinate when environmental conditions become favorable, thereby enhancing the adaptability of backcross offspring seeds to changing or unpredictable environments. In addition, backcrossing between cultivated and wild soybeans may alter the physical properties of the seeds of backcross progeny, including seed color, texture, and size, which may in turn affect seed dispersal. Nevertheless, this aspect has received relatively little research attention.

Although the Chinese government has not yet approved the commercial cultivation of GM soybean, several industrialization pilot projects have been launched since 2021 for herbicide-tolerant GM soybean lines that have obtained production and application safety certificates [29,30]. By 2024, the number of demonstration counties for industrialized pilot planting had increased to 47 counties (cities) across 7 provinces (regions), and all domestically developed insect-resistant and herbicide-tolerant GM soybean varieties with independent intellectual property rights and promising application prospects are expected to be included within the pilot planting scope [31]. These developments indicate that the commercial cultivation of GM soybean in China is imminent. Moreover, China imports substantial quantities of soybeans annually from countries such as the United States and Argentina, with a total import volume of 109 million tons in 2024, more than 90% of which consisted of GM soybeans [32]. Seed loss during transportation, storage, and processing may facilitate the establishment of feral populations in the wild [12,14,26,33]. Thus, the entry of GM soybean into Chinese farmland and natural ecosystems is inevitable, and it is therefore essential to evaluate the ecological risks of gene flow from GM soybeans to wild soybeans before they enter commercial production or large-scale planting in China. In this study, we established a backcross progeny system by repeatedly backcrossing resistant F_1_ plants with wild soybean plants. By assessing the expression of exogenous genes, seed dormancy, and the ecological fitness of backcross progeny, we evaluated whether the exogenous gene could persist and spread in wild soybean populations over the long term, as well as its potential impact on wild soybean genetic resources.

## 2. Results

### 2.1. Seed Germination and Dormancy

Significant differences in seed germination and dormancy were observed between GM soybean, wild soybean, and backcross progeny seeds (Table 1). GM soybean seeds exhibited the highest germination rate (93.05%) with a 0% dormancy rate, whereas wild soybean seeds showed the lowest germination rate (12.00%). The germination rates of F_1_, BC_1_F_1_, and BC_2_F_1_ seeds (34.57%, 37.12%, and 29.44%, respectively) were intermediate between those of GM and wild soybean seeds and differed significantly from both parental types. However, no significant differences in germination rate were detected between the F_1_ hybrid and backcross progeny seeds.

After the 21-day seed germination trial, the number of non-swollen seeds from the different materials was counted, and the seed dormancy was evaluated. The results showed that wild soybean, hybrid, and backcross progeny seeds exhibited a certain degree of dormancy. Among them, the germination rate of dormant JPW seeds reached 84.57%, which was significantly higher than those of the hybrid and backcross progeny seeds. The germination rates of dormant F_1_, BC_1_F_1_, and BC_2_F_1_ seeds ranged from 46.18% to 51.22%, with no significant differences among them. The specific germination rates of seeds from the different materials are presented in Table 1.

### 2.2. Herbicide Resistance and Exogenous Protein Expression Level 

Four days after glyphosate application, all of the JPW plants, 53.7% of the BC_1_F_1_ plants and 69.4% of the BC_2_F_1_ plants exhibited leaf wilting and yellowing, which was followed by plant death. In contrast, the GM and F_1_ plants, as well as the remaining 46.3% of BC_1_F_1_ and 30.6% of BC_2_F_1_ plants, showed no obvious phytotoxic response and continued normal growth. Leaf samples from these plants were subjected to ELISA for quantitative analysis. The results showed that the EPSPS protein was normally expressed in the GM, F_1_ hybrid, and backcross progeny populations.

**Figure 1:**
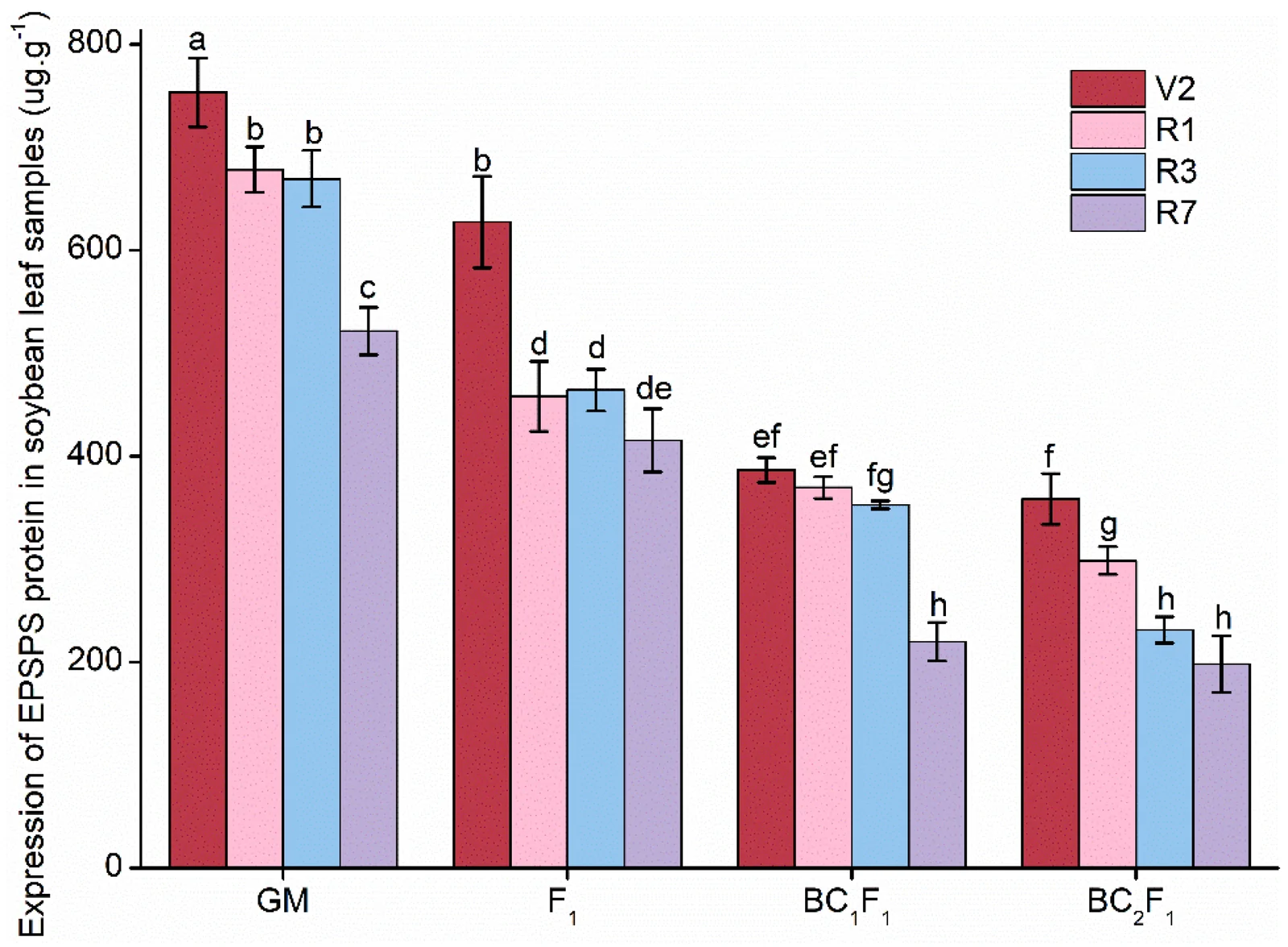
EPSPS protein expression levels in GM, F_1_ hybrid, and backcross progeny leaves. Values are means ± SD. Different lowercase letters indicate significant differences among treatments (Tukey’s test, *p* < 0.05).

EPSPS protein expression levels in the leaves of GM, F_1_, and BC_1_F_1_ plants varied between the growth stages: expression was relatively high at the V2 and R1 stages and decreased at the R3 and R7 stages. Across the different sampling stages, EPSPS levels in the leaves of GM plants ranged from 521.51 to 753.24 μg/g, whereas those in F_1_ and BC_1_F_1_ plants ranged from 415.40 to 627.49 μg/g and from 219.83 to 386.63 μg/g, respectively. EPSPS levels in BC_2_F_1_ plants were generally comparable with those in BC_1_F_1_ plants at all sampling stages. Moreover, at each growth stage, EPSPS expression in GM plants was significantly higher than that in hybrid and backcross progeny plants. The EPSPS protein expression levels in the leaves of the different plant materials at the various growth stages are shown in Figure 1.

### 2.3. Aboveground Biomass

**Figure 2:**
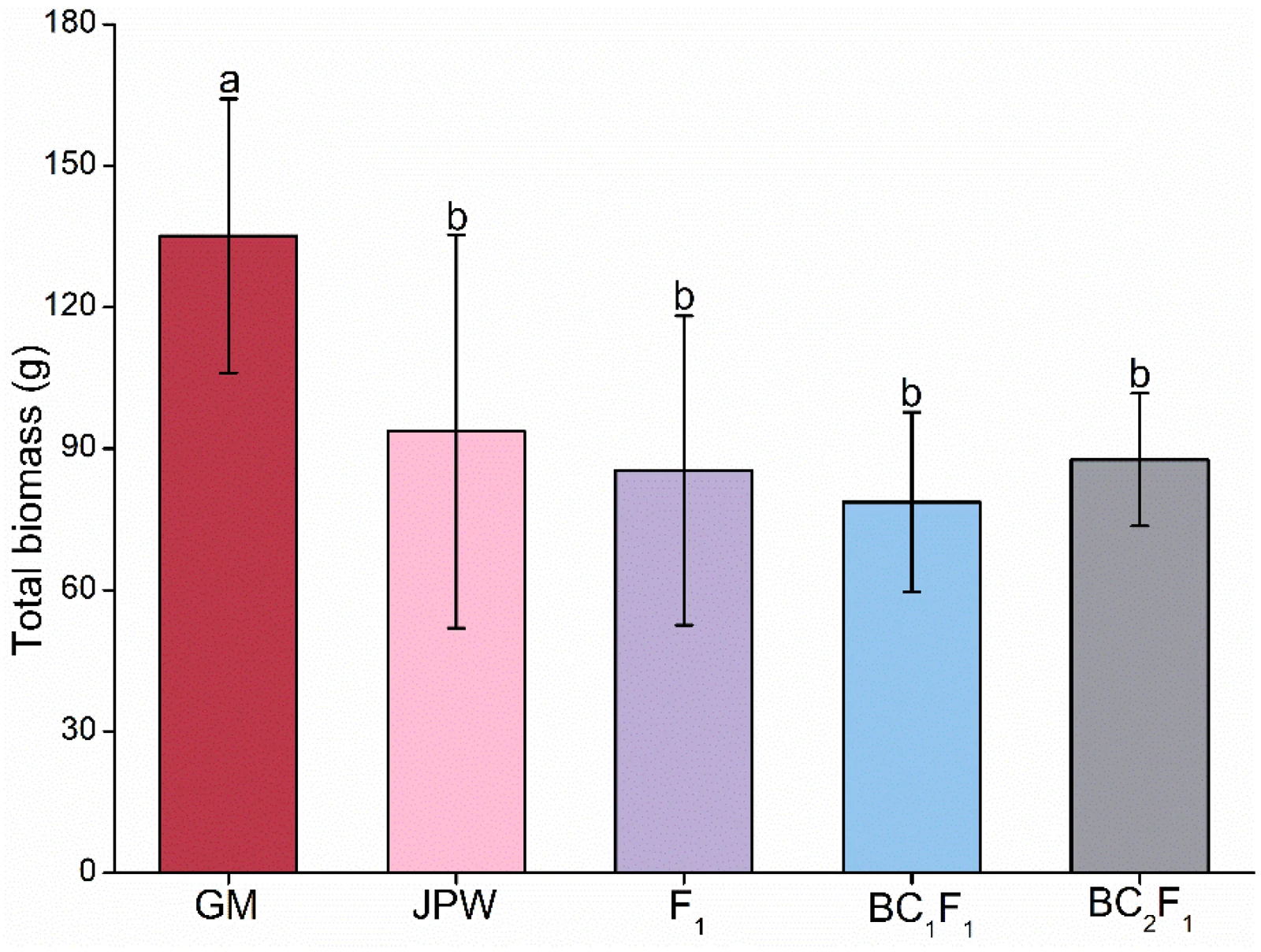
Aboveground biomass of GM, wild, F_1_ hybrid, and backcross progeny plants. Values are means ± SD. Different lowercase letters indicate significant differences among treatments (Tukey’s test, *p* < 0.05).

GM plants produced the highest aboveground biomass (135.13 ± 29.04 g), significantly greater than those for the F_1_, wild soybean, and backcross plants (Figure 2). The aboveground biomass of wild soybean plants was slightly higher than that of the F_1_, BC_1_F_1_, and BC_2_F_1_ plants, although the differences were not statistically significant (*p* > 0.05).

### 2.4. Yield Metrics

The number of pods per plant for the GM plants was significantly lower than those for the in F_1_ hybrid, backcross progeny, and wild soybean plants (Table 2). The average numbers of pods per plant for F_1_, BC_1_F_1_, and BC_2_F_1_ plants were 353, 450, and 521, respectively, all of which were significantly lower than that for wild soybean plants (*p* < 0.05). Compared with that for wild soybean, the number of pods per plant for BC_1_F_1_ plants decreased by 27.6%; however, no significant differences were observed between the F_1_ hybrid and backcross progeny plants.

In contrast, wild soybean, F_1_ hybrid, and backcross progeny plants produced more seeds than GM soybean plants. Specifically, the number of seeds per plant and the number of filled seeds per plant for GM soybean plants were 227 and 201, respectively, both significantly lower than those for the wild, F_1_ hybrid, and backcross progeny plants. Compared with wild soybean plants, the F_1_ hybrid and backcross BC_1_F_1_ and BC_2_F_1_ plants exhibited a certain degree of reproductive disadvantage: their seed number per plant and filled seed number per plant were significantly lower than those of wild soybean plants. Specifically, the number of seeds per plant and the number of filled seeds in F_1_ decreased by 34.30% and 36.9%, respectively, whereas those in BC_1_F_1_ decreased by 29.57% and 27.02%, respectively. BC_2_F_1_ plants showed smaller decreases than BC_1_F_1_ plants (16% and 18%, respectively). However, there were no significant differences between F_1_, BC_1_F_1_, and BC_2_F_1_.

Statistical analysis revealed that the 100-seed weight of the GM soybean plants was significantly higher than that of the wild soybean and hybrid/backcross progeny plants. The average 100-seed weight of F_1_ was 1.81 g, which did not significantly differ from that of the parental wild soybean plants. The 100-seed weights of BC_1_F_1_ and BC_2_F_1_ were 4.33 and 4.75 g, respectively, and were significantly greater than that of the parental wild soybean. However, there was no significant difference between BC_1_F_1_ and BC_2_F_1_.

## 3. Discussion

The introgression of resistance genes into wild relatives represents a key ecological risk associated with the release of GM crops, as it may increase the weediness of wild populations [34,35,36]. To date, most studies have focused on the ecological risks posed by direct pollen-mediated gene flow from GM crops into wild relatives. However, relatively few studies have addressed whether wild relatives that receive transgenic pollen may subsequently hybridize with other related species or evaluated the potential ecological risks associated with such secondary gene flow. In the present study, we assessed the ecological risks associated with pollen-mediated gene flow from F_1_ plants carrying resistance genes, thereby providing a scientific basis for the environmental safety assessment and sustainable utilization of GM soybeans.

### 3.1. Exogenous Protein Expression and Herbicide Resistance

ELISA analysis of leaf samples from BC_1_F_1_ and BC_2_F_1_ plants revealed that the exogenous EPSPS protein was stably expressed across the different sampling stages, although at significantly lower levels than in GM soybean plants. Consistently with our findings, Liu et al. [16] reported that the expression level of the exogenous CP4-EPSPS protein in BC_1_F_1_-positive backcross plants was comparable to that in the parental F_1_ plant and significantly lower than that in GM soybean plants, a conclusion also reached by Kubo et al. [37]. Although EPSPS expression levels decreased, herbicide resistance appeared to remain unaffected. After glyphosate application, no abnormalities were observed in backcross progeny plants carrying the *EPSPS* gene; this finding was consistent with those of other researchers [16,37]. Collectively, our results and those of previous studies indicate that once the exogenous gene escapes from GM soybean, it can gradually introgress into wild soybean populations through backcrossing and confer herbicide resistance in backcross progeny. Such gene flow from GM soybean to wild soybean populations may ultimately enable backcross progeny to evolve into superweeds.

### 3.2. Seed Dormancy and the Risk of Exogenous Gene Spread

The spread and persistence of exogenous genes in the natural environment depend largely on whether backcross progeny seeds exhibit dormancy. In this study, 84% of wild soybean seeds were dormant. In the germination trials, 46.18–51.22% of BC_1_F_1_, BC_2_F_1_ and F_1_ seeds retained dormancy while remaining viable. This dormant state extends seed longevity and thus enhances the environmental persistence of backcross progeny seeds. Although dormant seeds accounted for less than half of the total seeds used in the germination experiments, backcross progeny plants exhibited high seed yields. Upon seed maturation, pod dehiscence allowed many dormant seeds to disperse naturally into the soil, thereby markedly increasing the frequency with which seeds carrying exogenous genes entered the soil.

A previous study showed that the high dormancy of hybrid soybean seeds is closely associated with the seed coat, the impermeability of which to air and water represents a key factor inducing dormancy [38]. In this study, ungerminated seeds from backcross and hybrid progeny plants rapidly absorbed water and germinated once the constraints imposed by the seed coat were removed. Therefore, we hypothesize that seed dormancy in backcross progeny may also be associated with the seed coat. Future studies will be conducted to further elucidate the role of the seed coat in regulating seed dormancy in backcross progeny.

### 3.3. Ecological Fitness

In this study, the aboveground biomass of backcross progeny plants was slightly lower than that of wild soybean plants, indicating a slight reduction in vegetative growth capacity relative to the parental wild soybean. In contrast, the seed production traits of BC_1_F_1_ and BC_2_F_1_ plants were significantly inferior to those of wild soybean, with the number of pods per plant and number of seeds per plant being significantly lower than those of the parental wild soybean. Consistently with our findings, in a pot experiment with BC_1_F_1_ and BC_1_F_2_ populations derived from backcrossing the transgenic soybean GTS40-3-2 with the Zhengzhou wild soybean, Liu et al. [16] reported that the number of pods and filled seeds for BC_1_F_1_ plants were significantly lower than those for wild soybean. Although the pod numbers and filled seed number increased significantly for BC_1_ and BC_2_, they remained lower than those in wild soybean. Kuroda et al. [39] reached similar conclusions in a study of backcross progeny of cultivated soybean (Fukuyutaka) and wild soybean (JP110755). Liu [40] and Pandian et al. [41] also reported that the seed yield of BC_1_ seeds derived from backcrossing herbicide-tolerant transgenic rapeseed (*Brassica napus*) with wild mustard (*B. juncea*) was lower than that of wild mustard. The reduced yield of BC1 seeds may be attributed to factors such as the parental genetic background and reduced hybrid compatibility.

By comparing the growth potential, reproduction, and seed dormancy of parental plants and backcross progeny, the present study showed that first-generation backcross (BC_1_F_1_) plants derived from transgenic and wild soybean displayed vigorous vegetative growth, yet their seed yield was significantly lower than that of the wild soybean parent. This reduction may stem from genetic barriers between the F_1_ parent and the wild relative, as well as from reduced fertility associated with interspecific hybridization. In addition, seed dormancy in backcross progeny enhances their potential to spread within wild soybean populations once dormancy is broken, while the stable expression of exogenous genes confers herbicide resistance in backcross progeny, providing a substantial growth advantage under selection pressure. Notably, although the BC_1_F_1_ and BC_2_F_1_ progeny exhibited a certain reproductive disadvantage relative to wild soybean in this study, the fertility and fitness of backcross progeny may gradually recover after two or three generations of backcrossing [37,39]. Therefore, future research should evaluate the life-cycle performance of backcross progeny across successive generations under conventional and selective conditions, track the frequency and dynamics of the exogenous gene in wild populations over multiple generations under natural or simulated conditions (e.g., by monitoring the proportions of transgenic F_1_, F_2_, F_3_, BC_1_, and BC_2_ individuals), and examine the inheritance of the exogenous gene in F_1_, BC_1_F_1_, and BC_2_F_1_ progeny. Such experimental data would be interesting and useful, as they would not only reveal the evolutionary potential of the exogenous gene in wild populations but also enable the integration of data on gene flow, fitness, and inheritance patterns, thereby contributing to a more comprehensive understanding of gene escape, genetic introgression, and the associated ecological risks.

## 4. Materials and Methods

### 4.1. Experimental Materials and Cultivation Management

Two parents, namely, GM soybean GTS40-3-2 (referred to herein as GM) and Jiangpu wild soybean (referred to herein as JPW), were adopted in this study. Notably, GM soybean contains the *EPSPS* gene, which confers tolerance to the herbicide glufosinate, and was kindly provided by Nanjing Agricultural University (Nanjing, China). The GM soybean was generated via particle-bombardment transformation of soybean cultivar A5403, with an integrated functional cassette consisting of an enhanced CaMV 35S promoter, *cp4-epsps* coding sequence, CTP4 transit peptide, and NOS terminator. This transgenic event contains one full-length functional *cp4-epsps* copy and a non-functional 72 bp *cp4-epsps* fragment in its genome. Notably, no antibiotic resistance marker genes (*nptII*, *uidA*) were retained in the final genome, and glyphosate screening was used for transformant selection [42]. JPW seeds were collected in an agricultural field in Jiangpu County, Nanjing City, Jiangsu Province (N32°05′, E118°62′). All parental plants were grown in a greenhouse under controlled conditions (28 °C/22 °C day/night, 14 h photoperiod) with regular watering and fertilization. Healthy and uniformly growing plants were selected for artificial crossing experiments.

For the production of F_1_ progeny, the GM soybean was employed as the paternal parent, and JPW was chosen as the maternal parent. Emasculation was performed on unopened flower buds in the late afternoon one day before anthesis. The corolla and stamens were carefully removed with fine forceps without damaging the stigma. On the following morning (approximately 6:00–10:00 a.m.), fresh pollen was collected from fully opened flowers of GM and manually applied to the stigma of emasculated wild soybean flowers. Pollinated flowers were labeled with tags and bagged to prevent foreign pollen contamination. In total, 289 wild soybean maternal plants were used, and 7852 flower buds were hand pollinated. After maturation, a total of 174 F_1_ seeds were harvested.

In the following year, F_1_ seeds obtained the previous year were assessed for their germination rate, and the germinated seeds were sown and cultivated in the greenhouse. At the three-leaf stage, leaf samples were collected and QuickStix EPSPS transgenic detection test strips (EnviroLogix, Portland, ME, USA) were applied to screen for the presence of the transgene in F_1_. Samples presenting two clear detection lines (control line and test line) were identified as transgene-positive plants and used for subsequent backcrossing.

Following consistent pollination, cultivation, and molecular verification protocols, two successive backcross generations were constructed. For the first backcross, the positive F_1_ plant was used as the female parent and backcrossed with wild-type soybean, and 212 BC_1_F_1_ seeds were ultimately obtained. Verified glyphosate-positive BC_1_F_1_ plants were further backcrossed with wild soybean to produce BC_2_F_1_ populations, yielding 196 BC_2_F_1_ seeds. Seeds obtained from each generation were air-dried at room temperature and stored at 4 °C for subsequent analyses.

In each backcross experiment, all the plants were cultivated individually in pots containing farmland soil and potting soil (1/1 vol.) as the substrate (organic matter content of 2.8%). During the experiment, all the weeds in the pots were removed manually. In addition to regular watering, no fertilizers, chemical herbicides, or growth regulators were applied during the trial.

### 4.2. Trait Assessment

#### 4.2.1. Seed Dormancy Assay

For each material (F_1_, GM, JPW, BC_1_F_1_, and BC_2_F_1_), 150 seeds were randomly selected. The seeds were placed in 90 mm Petri dishes (Corning, NY, USA) lined with two layers of filter paper, which were moistened with distilled water. After labeling, the Petri dishes were incubated in a climate chamber (Binder KBF720, Tuttlingen, Germany) at 25 °C and 55% relative humidity under continuous darkness. The filter paper was replaced, and distilled water was added every 2 days. Germinated seeds, defined as those with a radicle length exceeding 5 mm, were counted and removed daily. Counting continued for 21 days, after which the daily seed germination rate was calculated.

Following the 21-day germination assessment, the remaining seeds were collected; all appeared intact, with no abnormalities in size, shape, or color. The seeds were then rinsed with distilled water, blotted dry with paper towels, and scarified by nicking the seed coat with a scalpel. Subsequently, a 5-day germination test was conducted as described above, and seeds that germinated after scarification were classified as dormant.

#### 4.2.2. Herbicide Resistance and ELISA Detection of the EPSPS Protein

Glyphosate tolerance was assessed by applying glyphosate to the leaves. At the third compound leaf stage, the selected leaves were wiped clean with a cotton swab dipped in a detergent solution. After the leaf surface had dried, a glyphosate solution (350 mg/L, Macklin Biochemical Co., Ltd., Shanghai, China) was applied evenly to the selected leaves, and leaf damage was recorded 3 days after treatment.

At the V2, R1, R3, and R7 growth stages [43,44], five plants that had survived glyphosate application were randomly selected from each material, and approximately 10 mg of leaf tissue was collected from each plant for a quantitative analysis of exogenous protein. A CP4-EPSPS ELISA kit (EnviroLogix) was used to quantify exogenous EPSPS protein levels in soybean leaves at the different growth stages, following the manufacturer’s instructions. After the assay, absorbance values were immediately measured at 450 nm using a microplate reader (IBM 2000, Armonk, NY, USA). Purified EPSPS protein was used as the quantitative standard, and the EPSPS protein content in the leaves was calculated based on the standard curve and the sample dilution factor.

#### 4.2.3. Growth and Yield Characteristics

Growth traits were analyzed for ten plants randomly selected from each soybean accession. At harvest, the plants were cut 1 cm above the ground and dried naturally, after which the aboveground biomass was determined using a PB602-N balance (Mettler Toledo, Greifensee, ZH, Switzerland). Yield-related traits, comprising seed weight per plant, number of pods per plant, number of filled pods per plant, number of seeds per plant, number of filled seeds per plant, and 100-seed weight, were then recorded.

#### 4.2.4. Data Analysis

Average and standard deviation values were calculated in SPSS version 20.0. Intergroup comparisons were conducted using one-way analysis of variance (ANOVA). Tukey’s multiple comparison test was employed to determine the significance of differences between groups. Moreover, *p* < 0.05 was considered to indicate statistical significance.

## Figures and Tables

**Table 1:** Mean proportions of germinated and dormant GM, wild, F_1_ hybrid, and backcross progeny seeds.

Plant Material	Germinated (%)	Dormant (%)
GM	93.06 ± 2.41 a	0
JPW	12.00 ± 3.61 c	84.57 ± 3.49 a
F_1_	34.57 ± 3.42 b	46.18 ± 4.53 b
BC_1_F_1_	37.12 ± 4.87 b	48.89 ± 5.12 b
BC_2_F_1_	29.44 ± 5.53 b	51.22 ± 4.16 b

Note: The data in the table are presented as the mean ± standard deviation. Different lowercase letters within the same column indicate significant differences among treatments at *p* < 0.05 (Tukey’s test, *p* < 0.05).

**Table 2:** Reproductive traits of GM, wild, F_1_ hybrid, and backcross progeny plants.

Plant Material	Pods per Plant	Seeds per Plant	Filled Seeds per Plant	100-Seed Weight (g)
GM	118 ± 31 c	227 ± 58 c	201 ± 54 c	18.92 ± 1.94 a
JPW	622 ± 104 a	1204 ± 275 a	992 ± 247 a	1.90 ± 0.05 c
F_1_	353 ± 136 b	791 ± 190 b	626 ± 174 b	1.81 ± 0.22 c
BC_1_F_1_	450 ± 80 b	848 ± 84 b	724 ± 85 b	4.33 ± 0.23 b
BC_2_F_1_	521 ± 45 b	986 ± 45 b	864 ± 21 b	4.75 ± 0.12 b

Note: The data in the table are presented as the mean ± standard deviation. Different lowercase letters within the same column indicate significant differences among treatments (Tukey’s test, *p* < 0.05).

## Data Availability

The data presented in this study are available on request from the corresponding author. The data are not publicly available due to institutional restrictions.
